# Microbial production of lactate-containing polyesters

**DOI:** 10.1111/1751-7915.12066

**Published:** 2013-05-29

**Authors:** Jung Eun Yang, So Young Choi, Jae Ho Shin, Si Jae Park, Sang Yup Lee

**Affiliations:** 1 Metabolic and Biomolecular Engineering National Research Laboratory, Department of Chemical and Biomolecular Engineering (BK21 Program), Center for Systems and Synthetic Biotechnology, KAIST291 Daehak-ro, Yuseong-gu, Daejeon, 305-701, Korea; 2 Institute for the BioCentury, KAIST291 Daehak-ro, Yuseong-gu, Daejeon, 305-701, Korea; 3 Department of Bio and Brain Engineering, Department of Biological Sciences, BioProcess Engineering Research Center, KAIST291 Daehak-ro, Yuseong-gu, Daejeon, 305-701, Korea; 4 Bioinformatics Research Center, KAIST291 Daehak-ro, Yuseong-gu, Daejeon, 305-701, Korea; 5 Department of Environmental Engineering and Energy (Undergraduate program), Myongji UniversitySan 38-2, Nam-dong, Cheoin-gu, Yongin-si, Gyeonggido, 449-728, Korea; 6 Department of Energy Science and Technology (Graduate program), Myongji UniversitySan 38-2, Nam-dong, Cheoin-gu, Yongin-si, Gyeonggido, 449-728, Korea

## Abstract

Due to our increasing concerns on environmental problems and limited fossil resources, biobased production of chemicals and materials through biorefinery has been attracting much attention. Optimization of the metabolic performance of microorganisms, the key biocatalysts for the efficient production of the desired target bioproducts, has been achieved by metabolic engineering. Metabolic engineering allowed more efficient production of polyhydroxyalkanoates, a family of microbial polyesters. More recently, non-natural polyesters containing lactate as a monomer have also been produced by one-step fermentation of engineered bacteria. Systems metabolic engineering integrating traditional metabolic engineering with systems biology, synthetic biology, protein/enzyme engineering through directed evolution and structural design, and evolutionary engineering, enabled microorganisms to efficiently produce natural and non-natural products. Here, we review the strategies for the metabolic engineering of microorganisms for the *in vivo* biosynthesis of lactate-containing polyesters and for the optimization of whole cell metabolism to efficiently produce lactate-containing polyesters. Also, major problems to be solved to further enhance the production of lactate-containing polyesters are discussed.

## Introduction

Chemicals, fuels and plastics produced by petroleum-based chemical and oil industry are widely used for the convenience of our daily life. Since such processes depending on fossil oil and gas are causing problems of resource depletion and climate change, there has been growing interest in producing chemicals, fuels and materials from renewable biomass through biorefinery processes. Microorganisms have been successfully employed as the key biocatalysts to produce chemicals, plastics and fuels from renewable resources. Since the development of the relatively recent industrial-scale biorefinery process for the production of 1,3-propanediol by Dupont and Tate & Lyle, several microorganisms have been metabolically engineered as potential platform biocatalysts for biorefineries to produce chemicals, fuels and materials. Some of these include butanol (Atsumi *et al*., 2008; Atsumi and Liao, 2008; Shen and Liao, 2008; Berezina *et al*., 2010; Jang *et al*., 2012a,b), isobutanol (Smith *et al*., 2010), higher alcohols (Zhang *et al*., 2008), diamines (Qian *et al*., 2009; 2011), aminocarboxylic acids (Park *et al*., 2012a; 2013), 1,4-butanediol (Yim *et al*., 2011), 3-hydroxypropionic acid (3HP) (Rathnasingh *et al*., 2009), lactic acid (Nguyen *et al*., 2012), succinic acid (Hong *et al*., 2004; Hong and Lee, 2004; Lee *et al*., 2006; Song and Lee, 2006), polylactic acid (PLA) (Jung *et al*., 2010; Yang *et al*., 2010a; 2011) and polyhydroxyalknoates (Taguchi and Doi, 2004; Reinecke and Steinbuchel, 2009; Gao *et al*., 2011; Park *et al*., 2012b,c)

More recently, systems metabolic engineering, which allows metabolic engineering at the systems-level by integrating systems biology, synthetic biology, protein engineering and evolutionary engineering, is enabling us to develop microorganisms more efficiently to produce various products, even including unnatural chemicals and materials that cannot be produced by employing the metabolic pathways and enzymes found in nature (Lee *et al*., 2012).

Polymers are essential materials in our daily life because they are light, durable, easy-to-make articles of interest, and relatively inexpensive. However, due to the problems mentioned earlier, there has been much interest in making polymers through biobased route using renewable biomass as a raw material. Some of the representative polymers produced through bioprocesses or combined biological-chemical processes include polyhydroxyalkanoates (PHAs), poly(butylene succinate) (PBS), poly(trimethylene terephthalate) (PTT), poly(lactic acid) (PLA) and nylons.

Biomass-derived polymers can be categorized largely into three groups. In the first group, polymers are entirely synthesized by biological processes, wherein microorganisms synthesize polymers using the monomers generated by inherent and engineered metabolic pathways of host strains from various carbon sources. Microbial fermentation results in direct synthesis of corresponding polymers that are accumulated in the host strains or are excreted into the culture medium. PHAs and poly(γ-glutamic acid) (PGA) are representative members of this group. The second group represents most of the currently produced biobased polymers where the polymer production process is a hybrid process combining both biological and chemical processes. All or some monomers and/or monomer precursors for polymers are produced by microbial fermentation, purified to polymer grade, and then are used for polymer synthesis. PBS, PTT, PLA and nylon 4 belong to this group. For example, 1,3-propanediol, one of the monomers for PTT, is produced by microbial fermentation and is used for copolymerization with petroleum-based terephthalate to synthesize PTT. On the other hand, homopolymers such as PLA and nylon 4 are composed exclusively of monomers produced by microbial fermentation. The present commercial process for the synthesis of PLA employs ring opening polymerization (ROP) of lactide, a dehydrated cyclic dimer of fermentation-derived lactic acid (Drumright *et al*., 2000; Sodergard and Stolt, 2002; Vink *et al*., 2003; Mehta *et al*., 2005). Nylon 4 is synthesized by ROP of 2-pyrrolidone, a dehydrated product of gamma aminobutyric acid (GABA), which is synthesized from glutamic acid (Liu *et al*., 2011a; Park *et al*., 2012a), one of the major amino acids produced by microbial fermentation. The third group is synthesized by complete chemical processes, in which polymers are chemically synthesized using monomers that are chemically derived from biomass. Nylon 5, 10 and nylon 6, 10 belong to this group, where sebacic acid, one of the monomers, is chemically derived from castor oil.

Among the biomass-derived and commercially available polymers, PLA has been one of the most attractive biobased polymers because of its biodegradability, biocompatibility, and compostability along with similar material properties compared with the general performance plastics. Several processes have been developed for the efficient production of PLA and its copolymers from renewable resources including ROP of lactide, which is now one of the commercially used processes. Although the current process for the production of PLA and its copolymers is a hybrid process in which the microbial processes for production of the monomers and the chemical processes for polymerization of the monomers are combined, the complete bioprocess for the synthesis of polymers containing lactate monomers has recently been developed by employing metabolically engineered microorganisms.

Here, we review recent advances in the production of lactate-containing polyesters by metabolically engineered bacteria. Metabolic engineering strategies for the design, construction and optimization of metabolic pathways for the development of recombinant microorganisms to efficiently produce lactate-containing polyesters are reviewed and discussed.

## Chemical synthesis of poly(lactic acid) (PLA)

### Chemical synthesis: ring opening polymerization (ROP)

**Figure 1:**
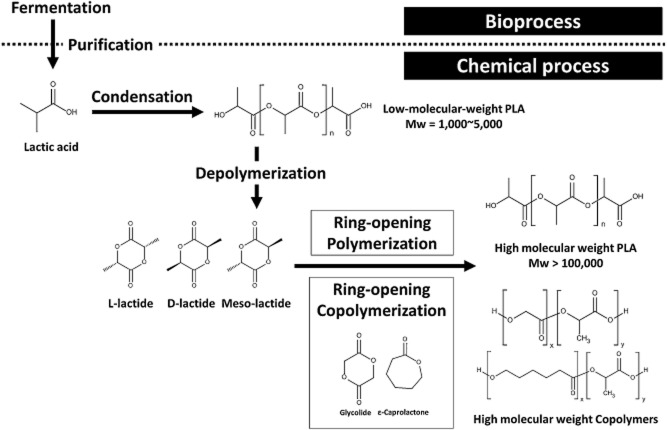
Chemical processes for production of high-molecular-weight PLA using fermentation-produced lactic acid as a starting material. The processes consist of three steps: condensation of lactic acid, depolymerization of low-molecular-weight PLA and ring-opening polymerization of lactides. Lactate containing copolymers, poly(lactate-*co*-glycolate) and poly(lactate-*co*-ε-caprolactone) can be produced by ring-opening copolymerization of lactide with glycolide and ε-caprolactone respectively. This figure is redrawn from a previous report (Vink *et al*., 2003).

Presently, NatureWorks produces PLA in a commercial scale of 140 000 tons year^−1^ using a bio and chemical hybrid process where microbial fermentation is used to produce lactic acid which is subsequently dehydrated to form the cyclic dimer, lactide (Vink *et al*., 2003). The lactide is then used for PLA synthesis by a metal-catalyst driven chemical process (Fig. 1).

Although the ring opening polymerization of lactide was first demonstrated by Carothers in 1932, high-molecular-weight PLAs could not be obtained until an improved lactide purification technique was developed by DuPont in 1954. The process of ROP for the production of PLA consists of three main steps (Fig. 1). First, lactic acid is condensed to give a low-molecular-weight PLA prepolymer (Mw = 1000∼5000). Second, the low-molecular-weight prepolymer is depolymerized to make lactide. In this process, a mixture of l-lactide, d-lactide and meso-lactide is created and the composition of the formed isomers depends on the isomer feedstock, reaction temperature, and the type of the catalyst. Finally, ROP of lactide is performed to make high-molecular-weight PLAs (Mw > 100 000). Since the fermentation product mainly consists of L-lactic acid with a small amount of d-lactic acid in the method used by NatureWorks, l-PLA is dominantly formed through ROP process. The characteristics of PLAs such as melting temperature, degree of crystallinity, mechanical properties and rate of degradation depend on the composition of the stereoisomers. In general, mono-isomeric PLAs possess higher melting points than PLAs consisted of mixed stereoisomers. In chemical process, a process for separating the stereoisomers is required (Vink *et al*., 2003).

Depending on the type of the initiator, cationic or anionic ROP processes can be used for PLA synthesis. However, these processes have drawbacks since they require high temperature and the side reactions hinder propagation. Additionally, the cationic ROP can lead to low-molecular-weight polymers and the anionic ROP can lead to racemization. Thus, ROP by coordination–insertion polymerization was developed by employing tin octanoate and aluminum alkoxides (Dubois *et al*., 1991; Gong and Ma, 2008). Since this modified ROP method allows production of higher-molecular-weight PLA with less risk of side reactions, NatureWorks recently has developed commercial process of PLA production employing this method.

### Copolymerization

PLA homopolymer has several undesirable material characteristics including poor rigidity, slow degradation rates and lack of reactive side-chain groups. To overcome such problems, many modifications on PLA have been explored, and one of the commonly used modification type is copolymerization. PLA has been copolymerized with various monomers such as glycolide, ε-caprolactone, 3-hydroxybutyric acid to produce copolymers either through polycondensation copolymerization or ring opening copolymerization (ROC) (Rasal *et al*., 2010). These two methods differ in that polycondensation copolymerization normally yields low-molecular-weight copolymers and ring opening copolymerization produces high-molecular-weight copolymers.

Polycondensation polymerization allows the condensation of the polyesters through the hydroxyl and acid groups present in the lactic acid molecule. Various monomers that also have such hydroxyl and acid groups can easily copolymerize with lactic acid *via* polycondensation without necessarily using a catalyst. An important advantage this method has is the control over polymer end groups. By reacting lactic acid with diols or diacids which have hydroxyl and acid functional groups, respectively, on both sides, the resulting copolymer can be chosen to have either hydroxyl or acid end groups. Additionally, controlling the concentrations of monomers leads to the production of polymers with controlled molecular weights. Despite the excellent control over the end groups, polycondensation copolymerization mainly produces low-molecular-weight polymers. This disadvantage can be overcome by using chain extenders such as diisocyanate to produce higher-molecular-weight polymers. Previously, hexamethylene diisocyanate (HDI) has been successfully used as a chain extender to synthesize thermoplastic elastomers (Zeng *et al*., 2009; Zhang *et al*., 2009).

As described above, ROP is a widely used method to make PLA from lactic acid. ROC is a common approach for the synthesis of PLA copolymers. Various transition metals such as aluminum (Dubois *et al*., 1991), tin (Kowalski *et al*., 2000), lead (Kricheldorf and Serra, 1985), zinc (Bero *et al*., 1990), bismuth (Kricheldorf and Serra, 1985), yttrium (Chamberlain *et al*., 2000) and iron (Stolt and Södergård, 1999) have been used to catalyse ROC (Rasal *et al*., 2010).

Lactic acid copolymerized with ethylene glycol has also been widely used because of biocompatibility and increased hydrophilicity of the resulting copolymer. The higher hydrophilicity of this copolymer prevents inflammatory responses from the living host upon direct contact with biological fluids when used in biomedical applications. Poly(lactate-*co*-ethylene glycol) copolymer is biocompatible and thus can be used in drug delivery systems with enhanced properties (Ben-Shabat *et al*., 2006; Jain and Kumar, 2010).

For such materials that require rapid degradation, the slow degradation rate of PLA has been improved by ROC of glycolide, l-lactide and ε-caprolactone with PLA (Rasal *et al*., 2010). The resulting copolymer poly(glycolate-*co*-lactate-*co*-ε-caprolactone) (PGLC) showed various degradation rates depending on its composition. For this case, copolymers having higher glycolate fraction gave higher degradation rates than those having higher lactate fraction (Cai *et al*., 2002).

## Non-chemical methods for production of polyesters containing lactate monomers

In the chemistry-based polyester synthesis that generally uses organometallic catalysts, it is impossible to completely remove the organometallic compounds from the polymers (Varma *et al*., 2005). Complete removal of such compounds is important especially for biomedical applications of the synthesized polymers. Therefore, metal-free organic catalysts have been drawing good attention for developing environmental-friendly and biocompatible plastics.

Enzyme catalysis has been suggested as a promising method for producing polymers of interest (Varma *et al*., 2005). Thus, extensive studies have been carried out for exploring the capacities of enzymes for producing various polymers including those that are not normally produced by using conventional chemical catalysts. The enzyme-catalysed polymerization offers several advantages compared with the chemical methods: (a) high enantio- and regio-selectivity can be achieved; (b) reactions proceed under mild temperature, pressure and pH conditions; (c) certain enzymes can be used in organic media; (d) complete removal of the enzymes from the reaction mixture after polymer synthesis is not strictly needed because enzymes are in general non-toxic; and (e) synthesized polymers have well-defined structures (Varma *et al*., 2005). However, the disadvantages that currently hinder actual usage of enzymes in the polymer industry are as follows: (a) high costs of the enzymes required for the reactions; (b) slow reaction rate; and (c) often yielding low-molecular-weight polymers that are not suitable for general applications (Varma *et al*., 2005).

One of the enzymes that have been extensively studied for polymer synthesis is lipase. Lipases naturally catalyse the hydrolysis of fatty acid esters. However, in organic solvents, lipases mediate polycondensation reactions. Thus, a method of lipase-catalysed PLA synthesis has been developed to replace the metal catalyst methods (Lassalle and Ferreira, 2008). Since this method of employing lipase cannot be used for *in vivo* polyester synthesis, other enzymes and pathways need to be recruited for fulfilling polyester synthesis *in vivo*.

### Biochemical pathways leading to polymer synthesis

**Figure 2:**
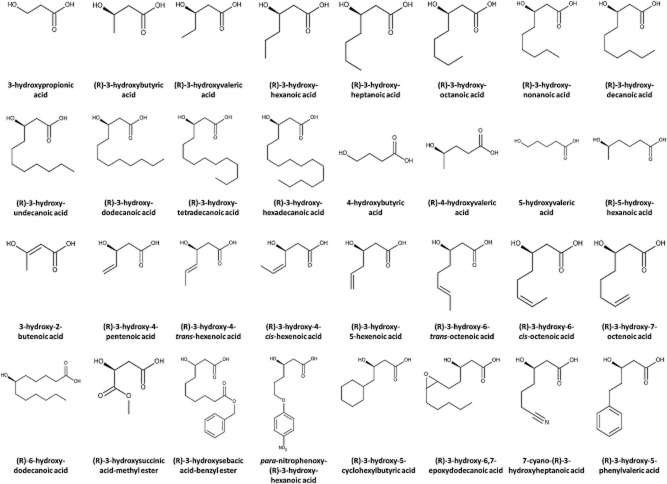
Exemplary molecules identified as PHA monomers in the microbial synthesis. The monomers differ by the carbon lengths, position of the hydroxyl groups and the functional groups attached. This figure is redrawn from a previous report (Steinbuchel and Valentin, 1995).

**Figure 3:**
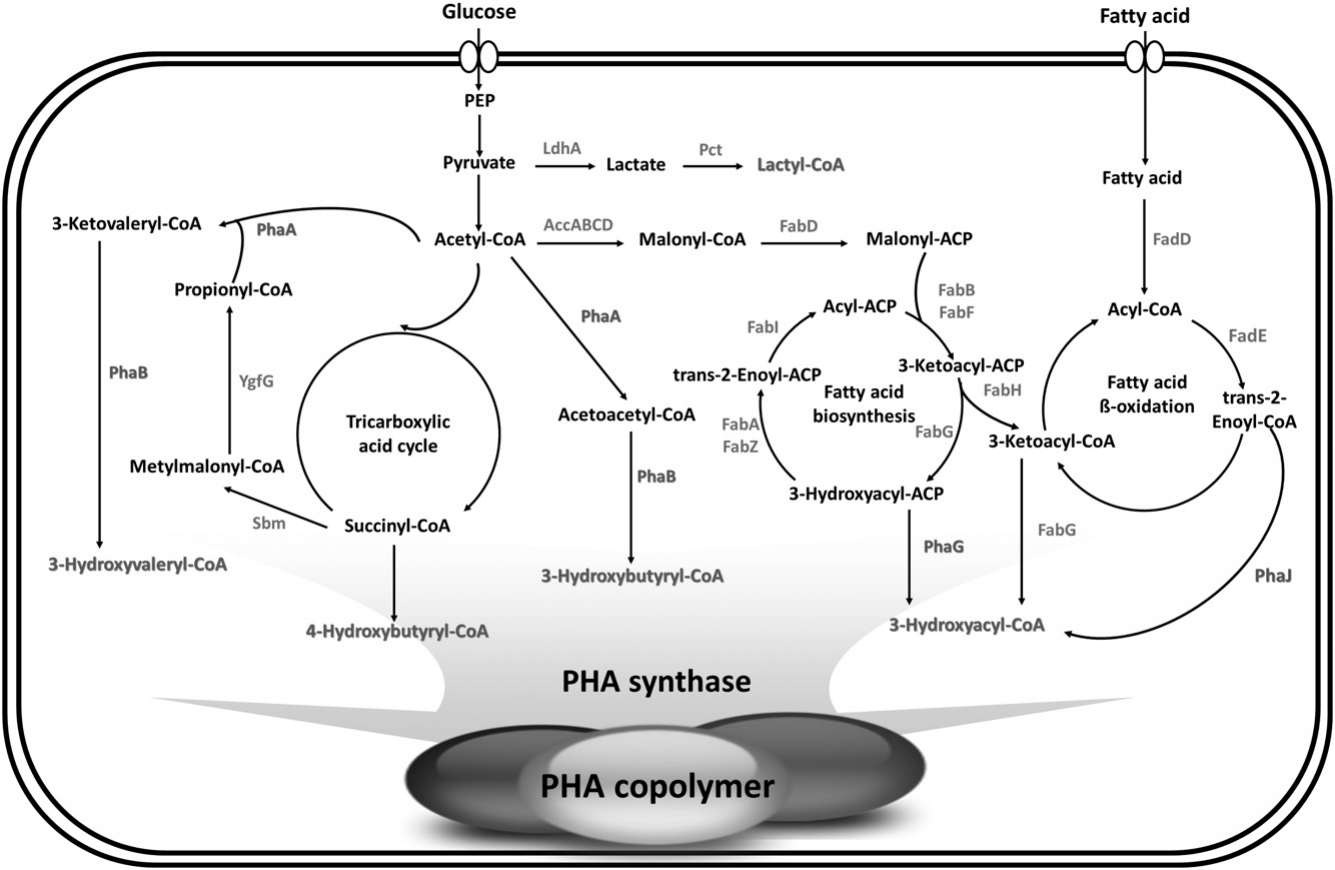
Various metabolic pathways for production of PHAs. Various hydroxyacyl-CoAs are accepted by the PHA synthase for polymerization. The major enzymes involved in PHA synthesis are shown. Abbreviations are: PhaA, β-ketothiolase; PhaB, acetoacetyl-CoA reductase; PhaG, (*R*)-3-hydroxyacyl ACP: CoA transacylase; PhaJ, (*R*)-specific enoyl-CoA hydratase; FadD, long-chain acyl-CoA synthetase; FadE, acyl-CoA dehydrogenase; FabG, 3-oxoacyl-ACP reductase; FabD, malonyl transferase; LdhA, lactate dehydrogenase; Pct, propionyl-CoA transferase; YgfG, methylmalonyl-CoA decarboxylase; Sbm, sleeping beauty mutase; AccABCD, acetyl-CoA carboxylase; FabB, F, H, 3-oxoacyl-ACP synthase I, II, III respectively; FabZ, (*R*)-3-hydroxymyristol ACP dehydratase; FabA, beta-hydroxydecanoyl thioester dehydrase; FabI, enoyl-ACP reductase; ACP, acyl carrier protein. This figure is redrawn by collecting information from previous reports (Aldor *et al*., 2002; Jung *et al*., 2010; Park *et al*., 2012c).

In nature, many microorganisms accumulate PHAs as carbon, energy and redox storage materials when they encounter unfavourable growth condition in the presence of excess carbon source. To date, more than 150 different kinds of 3-, 4-, 5- and 6-hydroxycarboxylic acids (Fig. 2) have been identified as monomer constituents of PHAs that are synthesized and accumulated as distinct granules in the cytoplasm of microorganisms (Steinbuchel and Valentin, 1995). Together with several key enzymes for the synthesis of PHAs from hydroxyacyl-CoAs, the metabolic pathways including glycolysis, TCA cycle, fatty acid β-oxidation and fatty acid biosynthesis are involved (Fig. 3). The key steps for PHA biosynthesis are as follows: generation of hydroxyacyl-CoA (HA-CoA) and polymerization of HA-CoAs into PHA by PHA synthase. When hydroxycarboxylic acids are generated in the cells, CoA is transferred to the hydroxycarboxylic acids to yield HA-CoAs that are required for PHA biosynthesis. The PHA monomer spectrum is quite broad with respect to the carbon numbers (3–16), the degree of saturation, different functional groups attached and the position of hydroxyl group (Fig. 2) (Steinbuchel and Valentin, 1995). Furthermore, PHA monomers are all in (*R*)-configuration, if a chiral centre exists on the carbon the hydroxyl group is attached to (Lee, 1996; Madison and Huisman, 1999). The material characteristics of PHAs are governed by the monomer constituents, and thus can be largely designed by metabolic engineering. However, among the various HA-CoAs synthesized in the host strain, only HA-CoAs that fit in the active site of PHA synthase can be incorporated to form PHAs.

In order to employ the PHA biosynthesis system as a platform for the *in vivo* synthesis of lactate-containing polyesters, two key pathway steps should be developed. First, host strains need to synthesize lactyl-CoA through inherent or heterologous metabolic pathways. Second, the PHA synthase needs to be engineered to accept lactyl-CoA as the substrate. However, until recently, no microorganisms have been identified to synthesize lactate-containing polyesters and no enzyme has been identified to polymerize lactyl-CoAs. This might be because the screened microorganisms lack either or both steps in the lactate-containing polyester synthesis, or because the screening effort has not yet been complete for identifying lactate-containing polyesters. Previously, the representative PHA synthases from *Ralstonia eutropha*, *Allochromatium vinosum* and *Ectothiorhodospira shaposhnikovii* have shown to possess negligible substrate specificities towards 2-hydroxyacyl-CoA such as lactyl-CoA and 2-hydroxybutyryl-CoA (Yuan *et al*., 2001; Zhang *et al*., 2001). However, the fact that PHA synthases show activities, although very low, towards lactyl-CoA, it was reasoned lactate-containing PHAs might be synthesized by employing engineered PHA synthases.

### Key enzymes for the biosynthesis of lactate-containing PHAs

Both the lactyl-CoA biosynthetic pathway and the PHA synthase that can accept lactyl-CoA as a substrate are required in order to complete the metabolic pathways for the production of lactate-containing PHAs in microorganisms. In alanine fermentation pathway existing in several microorganisms, such as *Clostridium propionicum*, *Megasphaera elsdenii*, *Bacteroides ruminicola* and *C. homopropionicum*, activation of (*D*)-lactate into (*D*)-lactyl-CoA plays an important role in the reductive branch of alanine fermentation pathway by accepting CoA from propionyl-CoA (Selmer *et al*., 2002).

Among various propionyl-CoA transferases (Pct) capable of catalysing this reaction, *C. propionicum* Pct has been explored in recombinant host strains such as *Escherichia coli* and the specific activity was also measured *in vitro* (Selmer *et al*., 2002; Cho *et al*., 2006; Yang *et al*., 2010a). Although it has been suggested that propionyl-CoA is the main CoA donor to activate (*D*)-lactate to (*D*)-lactyl-CoA in *C. propionicum*, acetyl-CoA can also be used as a CoA donor when the Pct is expressed in recombinant *E. coli*. *M. elsdenii* Pct has been employed for the construction of a metabolic pathway to produce 3-hydroxypropionic acid (3HP) using lactyl-CoA as a precursor for 3HP-CoA as described in the patent of Cargill (Gokarn *et al*., 2002). So far, the propionyl-CoA transferases from these two organisms have been actively employed for the *in vivo* generation of lactyl-CoA, and consequently for the synthesis of lactate-containing PHAs (Taguchi *et al*., 2008; Tajima *et al*., 2009; Yamada *et al*., 2009; 2010; 2011; Jung *et al*., 2010; Yang *et al*., 2010a,b; 2011; Park *et al*., 2012c,d).

Another key enzyme for the biosynthesis of lactate-containing PHAs is the PHA synthase, the enzyme responsible for the polymerization of lactyl-CoA. PHA synthase can be classified into four representative classes according to their subunit compositions and carbon numbers of their preferred substrates. The class I and II PHA synthases are dimers of PhaC subunits. The class I PHA synthases polymerize short-chain-length (SCL) HA-CoAs, while the class II PHA synthases generally show activities towards medium-chain-length (MCL) HA-CoAs except for some class II PHA synthases including those of *Pseudomonas* sp. 61-3 and *Pseudomonas* sp. 6–19 which can utilize both SCL- and MCL-substrates. The class III PHA synthases, composed of PhaC and PhaE subunits, are highly specific for SCL-HA-CoAs. The class IV PHA synthases are composed of PhaC and PhaR subunits and accept SCL-HA-CoAs as substrates (Park *et al*., 2012b). Representative class I, II, III and IV PHA synthases have been examined for PHA production both *in vivo* and *in vitro* employing recombinant *E. coli* and purified PHA synthase, respectively, where engineered Pct is also used in both cases for supplying lactyl-CoA (Taguchi *et al*., 2008; Tajima *et al*., 2009; Yang *et al*., 2010a; 2011). It was found that the activities of natural PHA synthases toward lactyl-CoA are not strong enough for the biosynthesis of lactate-containing PHAs. The use of class I PhaC from *R. eutropha* H16, class III PhaEC from *A. vinosum* DSM 180 and class IV PhaRC from *Bacillus cereus* ATCC 14579 allowed production of poly(3-hydroxybutyrate-*co*-lactate) having minute fraction of lactate monomer in recombinant *E. coli* depending on cultivation condition (Cho *et al*., 2006; Yang *et al*., 2010a). *In vitro* PHA synthesis system employing natural PHA synthases did not allow synthesis of lactate-containing polyesters (Taguchi *et al*., 2008; Tajima *et al*., 2009).

**Figure 4:**
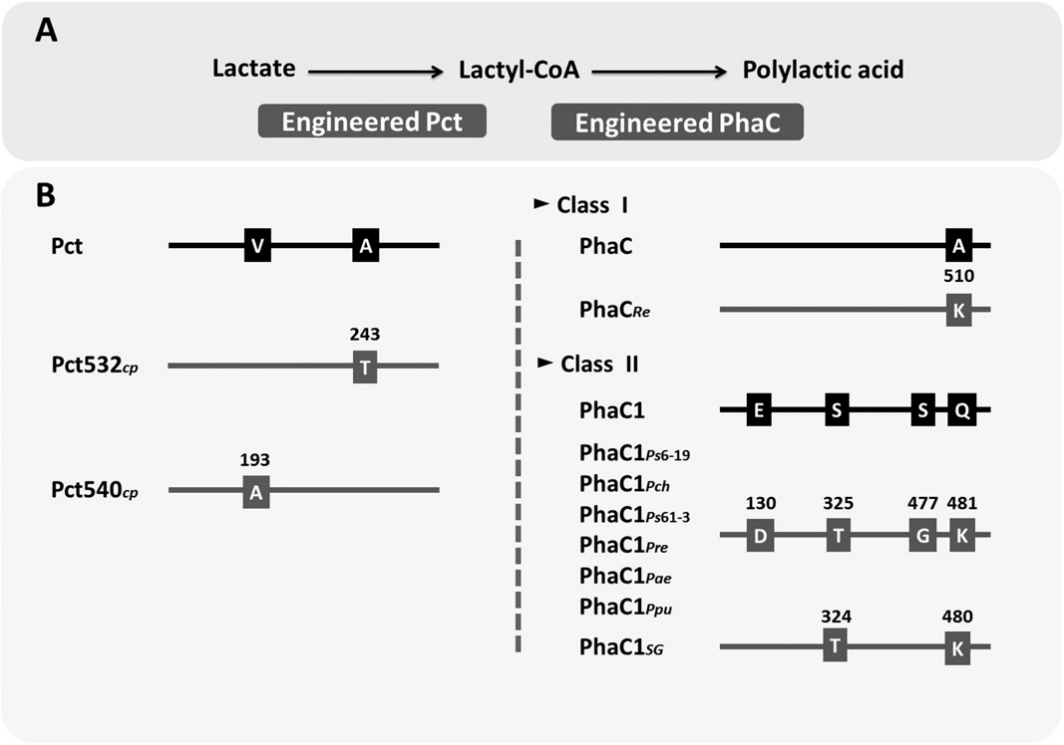
The key enzymes for biosynthesis of lactate containing polymers. A. In the PLA production pathway, the engineered propionyl-CoA transferase (Pct) converts lactate into lactyl-CoA. The lactyl-CoA is subsequently polymerized by the engineered PHA synthase (PhaC) to form PLA. B. Particular residues of the PHA synthesis enzymes are engineered to exhibit higher activities *in vivo*. The mutated sequences are shown in comparison with the wild type residues in the original polypeptides. Pct532_*cp*_, Pct540_*cp*_ are the mutants derived from the Pct of *C. propionicum* by error-prone PCR. Pct532_*cp*_ has an amino acid mutation (A243T) addition to the silent mutation (A1200G). Pct540_*cp*_ has four silent mutations of T78C, T669C, A1125G and T1158C addition to the V193A amino acid mutation. The PhaC_*Re*_, PhaC1_*SG*_, PhaC1_*Ps*6-19_, PhaC1_*Ps*61-3_, PhaC1_*Pch*_, PhaC1_*Ppu*_, PhaC1_*Pre*_ and PhaC1_*Pae*_ represent the mutants derived from PhaC1 of *Ralstonia eutropha* H16, *Pseudomonas* sp. SG4502, *Pseudomonas* sp. MBEL 6–19, *Pseudomonas* sp. 61-3, *Pseudomonas chlororaphis*, *Pseudomonas putida* KT2440, *Pseudomonas resinovorans* and *Pseudomonas aeruginosa* PAO1 respectively (Tsuge *et al*., 2004; Yang *et al*., 2011; Ochi *et al*., 2012; Tajima *et al*., 2012). In the original polypeptide sequence of PhaC1_*SG*_, S324 and Q480 corresponds to S325 and Q481 of PhaC1_*Ps*61-3_.

However, it is interesting to find that the engineered class II PhaC1 from *Pseudomonas* sp. MBEL 6–19 and *Pseudomonas* sp. 61-3, both of which have the combinatorial mutations in four amino acid residues (Glu130, Ser325, Ser477 and Gln481; Fig. 4), are able to accept lactyl-CoA as a substrate and allowed reproducible production of P(3HB-*co*-LA) (Taguchi *et al*., 2008; Shozui *et al*., 2009; Tajima *et al*., 2009; Yang *et al*., 2010a). It was suggested that these four amino acid residues (E130, S325, S477 and Q481) play important roles in conferring activity towards lactyl-CoA on PHA synthases. Thus, more thorough studies on the effects of mutations on these amino acid residues of representative class II PHA synthases from *P. chlororaphis*, *Pseudomonas* sp. 61-3, *P*. *putida* KT2440, *P*. *resinovorans* and *P*. *aeruginosa* PAO1 on the biosynthesis of lactate-containing PHAs were carried out (Yang *et al*., 2011). Site-directed mutagenesis of amino acid at 510, which corresponds to amino acid 481 of class II PHA synthase, was also carried out in class I PHA synthase from *R. eutropha* (Tsuge *et al*., 2004; Ochi *et al*., 2012). Furthermore, the residues S324 and Q480, which correspond to the residues S325 and Q481 of class II PHA synthase, were also mutated to threonine and lysine, respectively, in the thermostable PHA synthase from *Pseudomonas* sp. SG4502 (Tajima *et al*., 2012). Regardless of their class and source, all tested PHA synthases successfully synthesized P(3HB-*co*-LA) with different lactate mole fractions and molecular weights in recombinant *E. coli* or *in vitro* when they have desirable mutations in these amino acid residues (Yang *et al*., 2011; Ochi *et al*., 2012; Tajima *et al*., 2012).

### *In vivo* and *in vitro* methods for the synthesis of lactate-containing PHAs

For *in vitro* synthesis of lactate-containing PHA, water-organic solvent two-phase reaction system (TPRS) has been developed, in which 3HB-CoA and lactyl-CoA were continuously supplied to the PHA synthase after the ester exchange reaction between CoA and thiophenol where thiophenyl 3HB and thiophenyl lactate were donors (Han *et al*., 2009; Tajima *et al*., 2009). In the TPRS system, P(3HB-*co*-LA) was synthesized as a model polyester and was accumulated in the water-phase after the PHA synthase reaction (Tajima *et al*., 2009). Although the *in vitro* synthesis of P(3HB-*co*-LA) is suitable for examining enzyme characteristics involved in polymer synthesis, it has the following disadvantages for large-scale production: continuous feeding of substrates and separation of CoA molecules released after polymerization.

On the other hand, *in vivo* biosynthesis system allows one-step fermentation of engineered microorganisms for the production of lactate-containing polymer. Biosynthesis of P(3HB-*co*-LA) having different monomer fractions was achieved in recombinant *E. coli* expressing engineered Class II PHA synthase and Pct (Yang *et al*., 2010a). When *Pseudomonas* sp. MBEL 6–19 PhaC1s having combinatorial mutations in four E130, S325, S477 and Q481 sites were used along with engineered *C. propionicum* Pct, P(3HB-*co-*LA) copolymers having different monomer fractions, molecular weights and cellular contents were synthesized in recombinant *E. coli*. The highest mole fraction (49 mol%) of lactate monomer was achieved by using the quadruple mutant *Pseudomonas* sp. MBEL 6–19 PhaC1 having E130D, S325T, S477G and Q481K substitutions (Yang *et al*., 2010a). The molecular weights and thermal properties of P(3HB-*co*-LA) depended on the ratio of lactate to 3HB monomers. The higher lactate fraction in P(3HB-*co*-LA), the less molecular weight and the higher glass transition temperature the copolymer exhibited (Yang *et al*., 2010a).

## Metabolic engineering of microorganisms for the production of lactate-containing PHAs

### Recombinant *E. coli* for lactate-containing PHAs

Metabolic engineering strategies for the production of lactate-containing polyesters having different co-monomers have been developed using recombinant *E. coli* equipped with lactate-containing PHA biosynthetic pathway (Park *et al*., 2012b,c). *E. coli* has been used as a work horse for the production of a wide range of PHAs consisted of various hydroxycarboxylic acid monomers such as 3-hydroxypropionate (3HP), 3-hydroxyvalerate (3HV), 4-hydroxybutyrate (4HB), and a number of different medium-chain-length 3-hydroxycarboxylates having 6∼12 carbons including 3-hydroxyhexanoate (3-HHx) (Fig. 3).

For the production of additional classes of lactate-containing copolymers, various enzymes and engineered metabolic pathways supplying other classes of HA-CoAs have been explored: *C. acetobutylicum ptb* and *buk* genes encoding phosphotransbutylase and butyrate kinase for 4HB-CoA (Liu and Steinbuchel, 2000); *R. eutropha phaAB* genes encoding β-ketothiolase and acetoacetyl-CoA reductase for 3HV-CoA; *fadB* inhibition for MCL-monomers (Park and Lee, 2004; Liu *et al*., 2011b); engineered β-oxidation pathway for 3HV-CoA and 3HHx-CoA; and enoyl-CoA hydratase for 3HB-CoA and 3HHx-CoA (Shozui *et al*., 2010a; Kawashima *et al*., 2012). The strategies employed for these studies are covered in detail elsewhere (Park *et al*., 2012b,c). Among these studies, it is interesting to find that recombinant *E. coli* LS5218 produced terpolyester consisting of 96 mol% of lactate, 1 mol% of 3HB and 3 mol% of 3HV that shows PLA-like material properties due to very high fraction of lactate monomers. The lactate monomer fraction in terpolymer can also be increased up to 96 mol% with significant decrease of 3HB and 3HV monomer fractions by adding 0.4∼0.7 g l^−1^ sodium valerate to the culture medium (Table 1) (Shozui *et al*., 2011).

**Table 1:** Microbial production of lactate-containing polymers

			Molecular weight (Da)		
				
Host strain	Lactate containing polymer	PHA contents (wt%)	Mn (×10^4^)	Mw (×10^4^)	References
*Escherichia coli*	PLA	∼11	2.3	5.6	Jung *et al*. (2010); Yang *et al*. (2011)
	P(3HB-*co*-LA)	∼81	∼19	∼82	Shozui *et al*. (2010c); Yamada *et al*. (2011); Yang *et al*. (2011); Nduko *et al*. (2013)
	P(3HB-*co*-3HV-*co*-LA)	20–44	2.5–4.2	7.3–20	Shozui *et al*. (2010c)
	P(3HA*-*co*-LA)	6.3	–	2.7	Matsumoto *et al*. (2011)
	P(2HB-*co*-3HB-*co*-LA)	26.7–74.0	2	3.38	Park *et al*. (2012d)
*Ralstonia eutropha*	P(3HB-*co*-LA)	46.5	–	–	Yang *et al*. (2010b)
*Corynebacterium glutamicum*	P(3HB-*co*-LA)	1.4–2.4	0.43–0.52	0.57–0.74	Song *et al*. (2012)

3HB(3-Hydroxybutyrate); LA(lactate); 3HV(3-Hydroxyvalerate); 2HB(2-Hydroxybutyrate)

* 3HA includes 3-hydroxybutyrate, 3-hydroxyhexanoate, 3-hydroxyoctanoate, 3-hydroxydecanoate and 3-hydroxydodecanoate.

### Recombinant *E. coli* for PLA homopolymer

It is important to enhance the metabolic capacity of the host strain to supply enough precursors for increasing the PHA titre. Production of PHA copolymers enriched in specific monomer can be achieved by engineering the metabolic pathways to synthesize more of the particular precursor monomer (Fukui *et al*., 1999; Park *et al*., 2005). However, it was found to be challenging to develop recombinant *E. coli* for large-scale production of PLA homopolymer or the PHAs having enriched lactate monomer fraction since the substrate specificities of engineered PHA synthases towards lactyl-CoA are very low.

**Figure 5:**
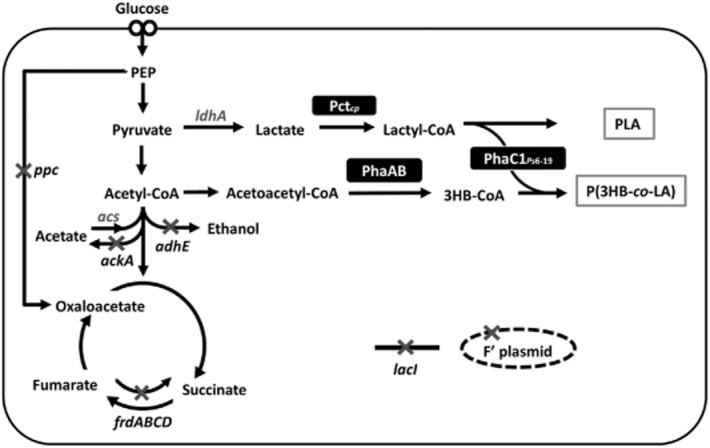
Systemic metabolic engineering of *Escherichia coli* XL1-Blue for enhanced production of PLA and P(3HB-*co*-LA) copolymer. The overall metabolic strategies are outlined in arrows. The cross marks represent chromosomal gene deletion for blocking competitive pathways. The *ldhA* and *acs* genes shown were overexpressed by chromosomal promoter replacement with the strong *trc* promoter. The F′ plasmid with *lacI*^*q*^ was eliminated from the host strain and the chromosomal *lacI* gene was also inactivated for constitutive expression of trc promoter induced genes. Abbreviations are: PhaA, β-ketothiolase; PhaB, acetoacetyl-CoA reductase; PhaC, PHA synthase; Pct, propionyl-CoA transferase; PEP, phosphoenolpyruvate. This figure is redrawn from the previous reports (Jung *et al*., 2010; Jung and Lee, 2011).

As a proof-of-concept study for PLA homopolymer synthesis in recombinant *E. coli*, metabolic pathways of *E. coli* XL1-Blue were engineered to increase lactyl-CoA synthesis (Jung *et al*., 2010). Since pyruvate is the direct precursor for lactate biosynthesis and acetyl-CoA is a CoA donor for lactyl-CoA synthesis, major competing pathways for these precursors were deleted. Additionally, lactate biosynthesis was improved by changing the native promoter of the *ldhA* gene with the strong *trc* promoter in the chromosome of *E. coli* XL1-Blue. Metabolic engineering strategies of *E. coli* XL1-Blue for the production of PLA homopolymer and other PHAs with enriched lactate monomers are depicted in Fig. 5. More specifically, the acetyl-CoA level was increased by deleting *ackA* and *adhE*, the genes in the competing pathways, and changing the native promoter of the *acs* gene in the chromosome of *E. coli* XL1-Blue with the strong *trc* promoter to enhance acetyl-CoA biosynthesis from acetate. Additionally, the *ppc* gene was deleted to increase lactate biosynthesis from pyruvate. When this *E. coli* JLX10 strain transformed with the plasmid for expressing the engineered class II PhaC1 from *Pseudomonas* sp. MBEL 6–19 and *C. propionicum* Pct, was cultured in a chemically defined medium containing 20 g l^−1^ of glucose, PLA homopolymer up to the polymer content of 11 wt% could be produced. Additionally, this strain also produced P(3HB-*co*-LA) having lactate monomer up to 86 mol% when 3HB was provided in the culture medium (Table 1) (Jung *et al*., 2010).

Since *E. coli* JLX10 strain needs IPTG for the induction of gene expression and succinate for cell growth because of *ppc* deletion, it is not desirable for the large-scale production of PLA homopolymer or lactate-enriched polymers. Thus, the metabolic pathways of *E. coli* XL1-Blue were re-engineered to overcome these disadvantages (Jung and Lee, 2011). First, the *trc* promoter in the chromosome can be constitutive by curing the F′ plasmid harbouring the *lacI^q^* gene and deleting the *lacI* gene in the chromosome. Second, in this strain, the *pflB*, *frdABCD* and *adhE* genes were completely deleted, and the native promoters of the *ldhA* and *acs* genes in the chromosome were replaced with the strong *trc* promoter to increase lactyl-CoA biosynthesis. When the final JLXF5 strain expressing the engineered class II PhaC1 from *Pseudomonas* sp. MBEL 6–19 and *C. propionicum* Pct was cultured in a chemically defined medium containing 20 g l^−1^ of glucose, PLA could be more efficiently produced compared with *E*. *coli* JLX10 (Jung and Lee, 2011). Fed-batch culture of JLXF5 expressing the engineered class II PhaC1 from *Pseudomonas* sp. MBEL 6–19, *C. propionicum* Pct and *R. eutropha* PhaAB allowed production of 20 g l^−1^ of P(60.4mol%3HB-*co*-39.6mol%LA) (Jung and Lee, 2011).

### Recombinant *R. eutropha* and *Corynebacterium glutamicum* for the production of lactate-containing PHAs

*Ralstonia eutropha* is one of the most efficient microorganisms for the production of PHAs, especially for the production of P(3HB) and P(3HB-*co*-3HV) from sugars (Jung and Lee, 2000; Reinecke and Steinbuchel, 2009). Additionally, P(3HB-*co*-3HHx) was produced by recombinant *R. eutropha* using fatty acid and oil as carbon sources in recent studies (Budde *et al*., 2011; Riedel *et al*., 2012). Due to the advantages of *R. eutropha* as the host strain for PHA production, such as fast growth in chemically defined media, multiple carbon sources being available for its cultivation, established genetic engineering tools, and well-developed high cell density culture techniques (Table 2), *R. eutropha* strains have been used for producing P(3HB) or P(3HB-*co*-3HV) in commercial scales by companies such as Biomer (Germany), Biocycle (Brazil), Telles (USA) and Tianan Biologic (China).

**Table 2:** Advantages and disadvantages of different host strains for the production of lactate-containing polymers

Strains	Advantages	Disadvantages	References
*Escherichia coli*	Fast growth	Products must be purified to get rid of endotoxin when used for biomedical applications	Kim *et al*. (2004); Matsumoto and Taguchi (2010); Shozui *et al*. (2010b,c)
	Established genetic engineering technology available		
	High-cell-density cultivation strategies available	Does not have endogenous genes for polymer production	
	Simple nutritional requisite		
*Corynebacterium glutamicum*	Endotoxin-free	Slow growth	Taguchi *et al*. (2008); Yamada *et al*. (2009)
	Extensive ability in assimilating crude sugar (i.e. molasses)	Low PHA contents in the engineered host	
*Ralstonia eutropha*	Naturally produces PHAs	Foam and emulsion formation when palm oil is fermented	Yamada *et al*. (2010); Park *et al*. (2012d)
	Fast growth		
	Multiple carbon sources available (i.e. palm oil and sugars)	Products must be purified to get rid of endotoxin when used for biomedical applications	
	Established genetic engineering technology available		
	High-cell-density cultivation strategies available		

For the production of lactate-containing PHAs by using recombinant *R. eutropha* NCIMB11599, the *phaCAB* operon involved in P(3HB) biosynthesis was completely replaced with the artificial operon consisting of the genes encoding PhaC1_*Ps*6-19_ and Pct (Yang *et al*., 2010b). Contrary to recombinant *E. coli* that efficiently produce (*D*)-lactic acid as a major fermentative product, recombinant *R. eutropha* did not efficiently produce (*D*)-lactic acid from glucose. Thus, lactic acid was added to the culture medium to support lactyl-CoA biosynthesis. Recombinant *R. eutropha* expressing engineered class II PHA synthase from *Pseudomonas* sp. MBEL 6–19 and *C. propionicum* Pct produced P(3HB-*co*-LA) containing 8.6 mol% LA with a polymer content of 24 wt% from 15 g l^−1^ glucose and 2 g l^−1^ lactate. It should be noted that removing the *phaAB* genes encoding β-ketothiolase and acetoacetyl-CoA reductase, both of which are mainly involved in the synthesis of 3HB-CoA from glucose, did not prevent 3HB monomers being incorporated in the polymer. It has been reported that many isoenzymes of PhaA and PhaB catalysing the same reactions exist in *R. eutropha* (Budde *et al*., 2010; Lindenkamp *et al*., 2012). If all of these isozymes can be removed without inhibiting cell growth and metabolism, PLA homopolymer synthesis in *R. eutropha* seems to be possible with strengthened lactyl-CoA biosynthetic pathway.

Since *Corynebacterium glutamicum* has been widely employed for industrial-scale amino acids production, there has been much interest to engineer it for the production of lactate-containing PHAs. P(3HB-*co*-LA) could be produced by expressing the engineered class II PHA synthase from *Pseudomonas* sp. 61-3 and *M. elsdenii* Pct (Song *et al*., 2012). The *E. coli ldhA* gene was also expressed to supply (*D*)-lactate for (*D*)-lactyl-CoA biosynthesis. Interestingly, P(3HB-*co*-LA) having very high lactate fraction (96.8 mol%) could be produced when the *R. eutropha phaAB* genes were expressed to supply more 3HB-CoA, the preferential substrate of PHA synthases. Without the expression of *R. eutropha phaAB* genes, the lactate monomer fraction in P(3HB-*co*-LA) was increased to 99.3 mol%, in which the polymer exhibited PLA-like characteristics. However, the polymer contents obtained by recombinant *C. glutamicum* were rather low (2.4 wt%), although cell concentration reached 12.7 g l^−1^, suggesting that further engineering and optimization of the metabolic pathways at the systems-level are needed for developing *C. glutamicum* as a platform strain for lactate-containing polyester production (Table 2) (Song *et al*., 2012).

## Extension of metabolic pathways for the production of other 2-hydroxycarboxylic acid containing PHAs

**Figure 6:**
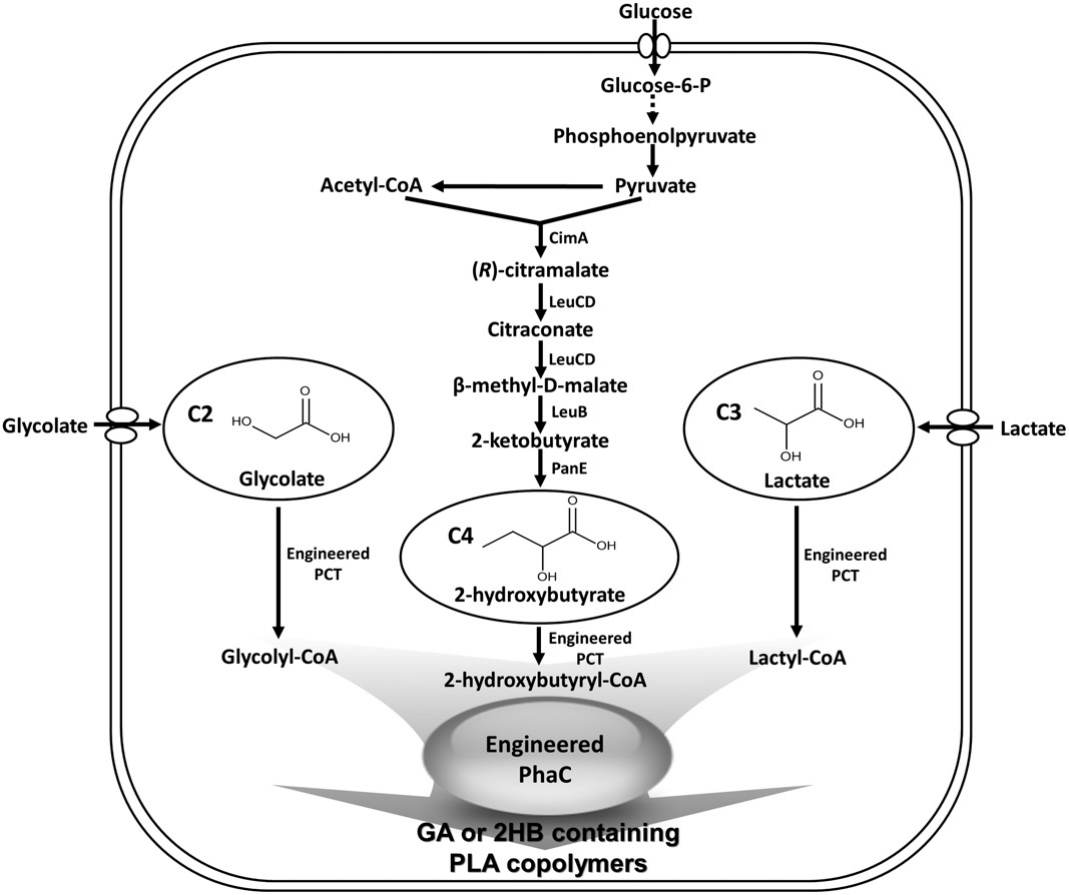
Extended metabolic pathways for production of the 2-hydroxyacid containing PHAs. Lactate-containing copolymers can be produced by engineered Pct and PhaC using lactate and glycolate (C2), lactate (C3) or 2-hydroxybutyrate (C4) as substrates. Although lactate feeding is shown in this figure, it is also possible to produce it *in vivo* as shown in Fig. 5. Metabolic pathways for production of PHAs containing 2HB monomers using glucose as the sole carbon source (Park *et al*., 2012d). Cross represents the competitive pathway blocked. Abbreviations are: CimA, citramalate synthase; LeuBCD, 3-isopropylmalate dehydratase; PanE, D-2-hydroxyacid dehydrogenase. This figure is redrawn from a previous report (Park *et al*., 2012d).

The versatile substrate specificities of engineered class II PHA synthases allow further extending the monomer spectrum of *in vivo* PLA synthesis system for the incorporation of glycolate, lactate and 2-hydroxybutyrate monomers into polyesters (Fig. 6). Glycolate, the simplest and shortest member of 2-hydroxycarboxylates, could also be incorporated into PHAs by engineered *Pseudomonas* sp. 61-3 PHA synthase when glycolate was supplied into the culture medium as a precursor (Matsumoto *et al*., 2011). Activation of glycolate and lactate to glycolyl-CoA and lactyl-CoA were mediated by *M. elsdenii* Pct, where both glycolyl-CoA and lactyl-CoA were used for the copolymerization with MCL-3-hydroxyalkanoyl-CoAs supplied by *P. aeruginosa* enoyl-CoA hydratase. The resulting copolymers containing glycolate and MCL-3-hydroxyalkanoates had weight-average molecular weight of 34 000 (Matsumoto *et al*., 2011).

Additionally, *E. coli* was engineered to produce PHAs containing 2-hydroxybutyrate (2HB) as a monomer from an unrelated carbon source (Park *et al*., 2012d). When the *ldhA*-deleted *E. coli* strain expressing the engineered *Pseudomonas* sp. MBEL 6–19 PHA synthase and *C. propionicum* Pct was cultured in a chemically defined medium containing 20 g l^−1^ of glucose along with different concentrations of 3HB and 2HB, PHAs containing 2HB, 3HB, and a small amount of lactate monomers were synthesized. The 2HB monomer fraction in copolymers varied from 10 mol% to 60 mol% depending on the concentrations of 2HB and 3HB added to the culture medium (Park *et al*., 2012d).

The metabolic pathway for the enhanced production of 2HB-CoA was also established in *E. coli* for the incorporation of 2HB in the copolymer without extracellular feeding of 2HB. The citramalate pathway consisting of *Methanococcus jannaschii* citramalate synthase (CimA) and *E. coli* LeuBCD were used for supplying 2-ketobutyrate (2KB) and *Lactococcus lactis* ssp. *lactis* Il1403 D-2-hydroxyacid dehydrogenase (PanE) for converting 2KB into 2HB (Park *et al*., 2012d). When the engineered *Pseudomonas* sp. MBEL 6–19 PHA synthase and *C. propionicum* Pct were expressed in recombinant *ldhA*-deleted *E. coli* strain equipped with citramalate pathway and PanE, PHAs consisted of 2HB, 3HB and a small amount of lactate monomers were produced by cultivation in a chemically defined medium containing 20 g l^−1^ of glucose and varying amount of 3HB. Additional expression of *R. eutropha phaAB* genes in this strain resulted in the production of P(2HB*-co-*3HB*-co-*LA) from glucose as a sole carbon source. However, the significant decrease of 2HB monomer fraction in the copolymer was observed. This is possibly because *R. eutropha* β-ketothiolase and acetoacetyl-CoA reductase used most of the acetyl-CoA molecules for the synthesis of 3HB-CoA, instead of synthesizing 2HB-CoA, which is derived from acetyl-CoA and pyruvate (Park *et al*., 2012d).

## Future perspectives

It is now possible to produce various lactate-containing polyesters by one-step fermentation of recombinant microorganisms equipped with the engineered PLA biosynthesis system. When we consider the key reactions for the biosynthesis of lactate-containing PHAs in recombinant microorganisms, two major problems should be solved for *in vivo* PLA biosynthesis system to become more competitive. The first major problem is related with lactyl-CoA biosynthesis using acetyl-CoA as a CoA donor. It is not an efficient way in a metabolic perspective for three reasons. First, consumption of acetyl-CoA for lactyl-CoA synthesis is not preferred for optimized cell growth since it is one of the most important central metabolites used in diverse cellular metabolism. Second, conversion of pyruvate into both acetyl-CoA and lactate hinders maximizing the lactyl-CoA pool, which ultimately affects PLA biosynthesis efficiency. Third, acetate is produced when using acetyl-CoA as a CoA donor for lactyl-CoA synthesis. Acetate, at high concentration, is known to be detrimental to cell growth. Indeed, it was found to be a problem in fed-batch culture for the high-level production of lactate-containing polyesters (Jung and Lee, 2011).

The second major problem is the extremely low activities of engineered PHA synthases to polymerize lactyl-CoA. Although certain engineered PHA synthases from *Pseudomonas* sp. and *R. eutropha* are able to synthesize lactate-containing PHAs in *E*. *coli*, the activities of these PHA synthases towards lactyl-CoA seem to be very low (Yang *et al*., 2010a) compared with those towards 3HB-CoA. Thus, it is necessary to further increase the activities of PHA synthases toward lactyl-CoA to a comparable level of high-performance *R. eutropha* PHA synthase that uses 3HB-CoA as a substrate. Since protein engineering of PHA synthase based on the exact structure is not currently possible, directed evolution and random mutagenesis of PHA synthase are useful methods to screen PHA synthases with enhanced polymerization activity towards lactyl-CoA.

Once these two major problems are solved by successful enzyme engineering, further engineering of the whole-cell metabolism can be performed to maximize the production of lactate-containing polymers in a cost-effective manner. Systems metabolic engineering strategies successfully demonstrated for the enhanced production of a wide range of chemicals and materials, and even for the construction of non-natural created pathways (Park *et al*., 2008; Na *et al*., 2010; Lee *et al*., 2012) will be useful for the development of the truly versatile and powerful platform strains for the production of various lactate-containing polyesters.
